# Copy Number Variation Analysis in Single-Suture Craniosynostosis: Multiple Rare Variants Including *RUNX2* Duplication in Two Cousins With Metopic Craniosynostosis

**DOI:** 10.1002/ajmg.a.33557

**Published:** 2010-08-03

**Authors:** Heather C Mefford, Neil Shafer, Francesca Antonacci, Jesse M Tsai, Sarah S Park, Anne V Hing, Mark J Rieder, Matthew D Smyth, Matthew L Speltz, Evan E Eichler, Michael L Cunningham

**Affiliations:** 1 Division of Genetic Medicine, Department of Pediatrics, University of WashingtonSeattle, Washington; 2 Department of Genome Sciences, University of WashingtonSeattle, Washington; 3 Department of Environmental and Occupational Health Science Functional Genomics Laboratory, University of WashingtonSeattle, Washington; 4 Division of Craniofacial Medicine, Department of Pediatrics, University of WashingtonSeattle, Washington; 5 Seattle Children's Craniofacial CenterSeattle, Washington; 6 Department of Neurosurgery & St. Louis Children's Hospital, Washington UniversitySt. Louis, Missouri; 7 Department of Psychiatry and Behavioral Science, University of WashingtonSeattle, Washington; 8 Howard Hughes Medical Institute, University of WashingtonSeattle, Washington

**Keywords:** craniosynostosis, copy number variant, array comparative genomic hybridization, *RUNX2*

## Abstract

Little is known about genes that underlie isolated single-suture craniosynostosis. In this study, we hypothesize that rare copy number variants (CNV) in patients with isolated single-suture craniosynostosis contain genes important for cranial development. Using whole genome array comparative genomic hybridization (CGH), we evaluated DNA from 186 individuals with single-suture craniosynostosis for submicroscopic deletions and duplications. We identified a 1.1 Mb duplication encompassing *RUNX2* in two affected cousins with metopic synostosis and hypodontia. Given that RUNX2 is required as a master switch for osteoblast differentiation and interacts with TWIST1, mutations in which also cause craniosynostosis, we conclude that the duplication in this family is pathogenic, albeit with reduced penetrance. In addition, we find that a total of 7.5% of individuals with single-suture synostosis in our series have at least one rare deletion or duplication that contains genes and that has not been previously reported in unaffected individuals. The genes within and disrupted by CNVs in this cohort are potential novel candidate genes for craniosynostosis. © 2010 Wiley-Liss, Inc.

## INTRODUCTION

Craniosynostosis is defined as the premature fusion of one or more cranial sutures and has an overall prevalence of 3–5 per 10,000 individuals [French et al., 1990; Cohen, 2000; Boulet et al., 2008]. Approximately 5–15% of craniosynostosis cases involve multiple cranial sutures, referred to as complex craniosynostosis. The remainder and vast majority (85–95%) of craniosynostosis cases are simple or single-suture craniosynostosis. Craniosynostosis can be further described as syndromic, in which other features are present in the patient, or non-syndromic. Several genes have been identified in which mutations cause syndromic forms of craniosynostosis. These include: *FGFR1*, *FGFR2*, *FGFR3*, *TWIST1*, *MSX2*, *EFNB1*, *TGFBR1*, *TGFBR2*, *FBN1*, *RECQL4*, *RAB23*, and *POR* [Passos-Bueno et al., 2008]. However, little is known about the genetics of the more common isolated non-syndromic forms of craniosynostosis. There are limited reports of mutations in *FGFR1*, *FGFR2*, *FGFR3*, and *TWIST1* [Renier et al., 2000; Mulliken et al., 2004; Seto et al., 2007] in some patients with non-syndromic single-suture craniosynostosis, but these explain only a small fraction of affected individuals.

Recently, copy number variants (CNVs; deletions, duplications) have been identified as an important source of mutation contributing to abnormal phenotypes. With the increasing availability and use of array comparative genomic hybridization (CGH) and SNP microarrays, now there are many examples of phenotypes that have been studied to evaluate for CNVs. In this study, we hypothesize that rare CNVs in patients with isolated single-suture craniosynostosis contain genes important for the phenotype. We evaluated DNA from 186 individuals with single-suture craniosynostosis for submicroscopic deletions and duplications using whole-genome array CGH in an effort to identify novel candidate genes for craniosynostosis. We identified 7.5% of individuals with rare deletions or duplications that contain genes and that have not been previously reported in unaffected individuals. Of these, one pair of cousins, each with metopic craniosynostosis, share a heterozygous ∼1 Mb duplication of 6p21 that encompasses *RUNX2* and is likely pathogenic. We identified several additional rare CNVs that may harbor candidate genes for craniosynostosis.

## METHODS

### Description of Cohort

Participants were enrolled after informed consent in a previously described, prospective, four-center investigation of neurodevelopment among children with single-suture craniosynostosis and children without prematurely fused sutures [Speltz et al., 2007]. Case infants were eligible for the larger study (the Infant Learning Project), if at the time of enrollment, they had isolated sagittal, unilateral coronal, metopic, or unilateral lambdoid synostosis confirmed by CT scan; had not yet had reconstructive surgery; and were ≤30 months of age. Cases were excluded due to premature birth (i.e., before 34 weeks gestation); presence of major medical or neurological conditions (e.g., cardiac defects, seizure disorders, cerebral palsy, significant health conditions requiring surgical correction, etc.); presence of three or more extra-cranial minor malformations [Leppig et al., 1988]; or presence of major malformations. We obtained independent institutional approval from each participating center: Seattle Children's Hospital (Seattle, WA); Northwestern University in Chicago (Chicago, IL), Children's Heath Care of Atlanta (Atlanta, GA), and St. Louis Children's Hospital (St. Louis, MO).

Infants were referred to the study at the time of diagnosis by their treating surgeon or pediatrician. Enrolled cases in the overall study were 84% of those eligible, with distance or time constraints being the major reason for non-participation. CT scans were performed at each participating center, and de-identified imaging data were sent to Seattle for further diagnosis confirmation. Neurodevelopment was evaluated with the Bayley Scales of Infant Development—2nd Edition [Bayley, 1993]. Prior to enrollment in this study all cases were screened for hot spot mutations in *FGFR1*, *FGFR2*, *FGFR3*, *TWIST1*, *MSX2* [Seto et al., 2007], and *EFNB1* and excluded if a causative mutation was identified.

### Array CGH

Array CGH was performed using a whole-genome tiling array with 135,000 oligonucleotide probes spaced approximately every 25 kb across the genome (Human CGH 12 × 135 k WG-T array, Roche NimbleGen, Inc, Madison, WI). Hybridizations were performed as previously described [Selzer et al., 2005]. Data are analyzed according to manufacturer's instructions using NimbleScan software to generate normalized log_2_ fluorescence intensity ratios. Then, for each sample, normalized log intensity ratios are transformed into z-scores using the chromosome-specific mean and standard deviation. z-scores are subsequently used to classify probes as “increased,” “normal,” and “decreased” copy-number using a three-state Hidden Markov Model (HMM). The HMM was implemented using HMMSeg [Day et al., 2007] which assumes Gaussian emission probabilities. The “increased” and “decreased” states are defined to have the same standard deviation as the “normal” state, but with mean z-score two standard deviations above and below the mean, respectively. Probe-by-probe HMM state assignments are merged into segments according to the following criteria: consecutive probes of the same state less than 50 kb apart are merged, and if two segments of the same state are separated by an intervening sequence of ≤5 probes and ≤10 kb, both segments and intervening sequence are called as a single variant. CNV calls are filtered to eliminate (i) events containing <5 probes and (ii) common CNVs that are also found in unaffected individuals. Filtered CNVs are also visually inspected in a genome browser. All events reported here were validated using a second, independent, higher-density oligonucleotide array platform (NimbleGen 2.1 M WG-T, NimbleGen 3 × 720 K Exon-Focused Array, custom NimbleGen 12-plex array described elsewhere [Mefford et al., 2009], or Agilent 1 M).

### Fluorescence In Situ Hybridization

Metaphase spreads were obtained from osteoblast cell lines. FISH experiments were performed using the fosmids WIBR2-2758A13 and WIBR2-1695P15, directly labeled by nick-translation with Cy3-dUTP and Fluorescein-dUTP essentially as previously described [Lichter et al., 1990], with minor modifications. Briefly, 300 ng of labeled probe was used for the FISH experiments, hybridization was performed at 37°C in 2× SSC, 50% (v/v) formamide, 10% (w/v) dextran sulphate, 5 µg COT1 DNA (Roche), and 3 µg sonicated salmon sperm DNA, in a volume of 10 µl. Posthybridization washing was at 60°C in 0.1× SSC (three times, high stringency). Nuclei were simultaneous DAPI stained. Digital images were obtained using a Leica DMRXA2 epifluorescence microscope equipped with a cooled CCD camera (Princeton Instruments, Trenton, NJ). DAPI, Cy3, and fluorescein fluorescence signals, detected with specific filters, were recorded separately as gray scale images. Pseudocoloring and merging of images were performed using Adobe Photoshop software.

## RESULTS

### Array CGH

**FIG. 1:**
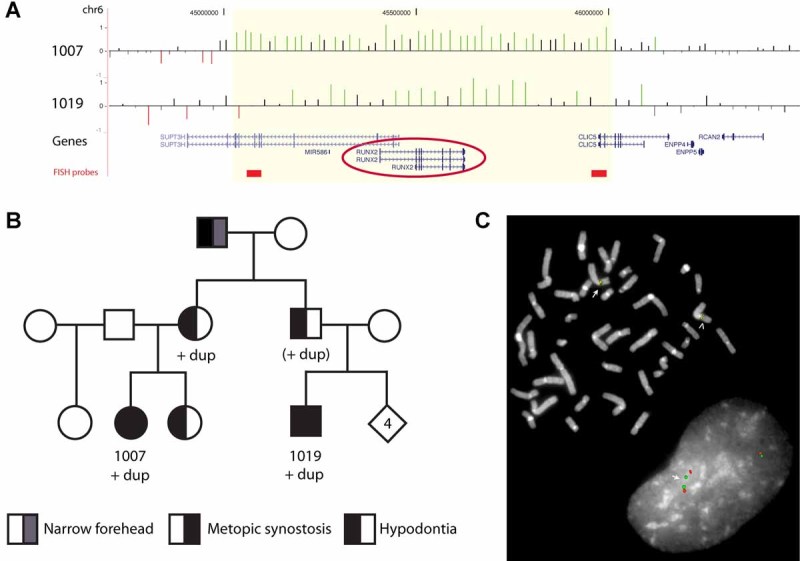
Duplication of chromosome 6p21 encompassing *RUNX2* in two affected cousins. **A**: Oligonucleotide array CGH results for cases 1007 and 1019 for chr6:44,700,000–46,500,000 (NCBI Build 36). For each individual, deviations of probe log_2_ ratios from zero are depicted by gray/black lines, with those exceeding a threshold of 1.5 standard deviations from the mean probe ratio colored green and red to represent relative gains and losses, respectively. Yellow shading represents extent of duplication based on follow-up high-density array CGH validation. Red bars show the mapped location of fosmids used for FISH. **B**: Pedigree showing relationship of cases 1007 and 1019 and phenotypic features. The father of 1019 is a presumed carrier of the duplication but DNA was not available for analysis. **C**: FISH analysis using two fosmid probes shows that the duplication is tandem (see metaphase spread, upper left) and inverted (see interphase nucleus, lower right). Results are shown for patient 1019; similar results were obtained for patient 1007. White arrows indicate chromosome 6 homolog carrying the inverted duplication. The normal chromosome 6 in the metaphase spread is indicated by the “>.”

We evaluated DNA from 186 individuals with single-suture craniosynostosis using a whole-genome oligonucleotide array CGH platform. We identified 14 individuals (7.5%) with at least one CNV that contained one or more genes and that has not been previously identified in 2,493 control individuals [Itsara et al., 2009] (Table I). The rare CNVs identified range from 35 kb to 3.9 Mb. Three patients (1.6%) harbored events >2 Mb compared to 8/2,493 controls (0.3%). DNA from one or more parents was available for analysis in seven cases. For four individuals, we confirmed that the CNV was inherited from a reportedly normal parent. In one case (4038), the deletion is absent in the mother, but DNA from the father was unavailable. For patient 1019 harboring a duplication of the *RUNX2* gene, inheritance is presumed to be paternal given the presence of the same duplication in a first cousin and paternal aunt (see below; Fig. 1); the duplication was not found in the mother, as expected. Parents were unavailable in the remaining seven cases. Case descriptions for individuals harboring events >1 Mb are below.

**TABLE I:** Rare CNVs Identified in 186 Individuals With Single-Suture Craniosynostosis

Sample number	Change detected	Coordinates (NCBI Build 36)	Size (Mb)	Inheritance	Candidate genes	Suture	Mental Dev Index^c^	Psychomotor Dev Index^c^
4038	9q22 del	Chr9: 93.59–97.51 Mb	3.92	Unk	*ROR2*, *ECM2*	M	SD	SD
1056	3p25 dup	Chr3: 11.96–15.30 Mb	3.34	Inh (P)	*FBLN2*, *TMEM43*	S	WNL	WNL
2082	5p15 dup	Chr5: 7.59–10.06 Mb	2.47	Inh (M)	*SEMA5A*, *FASTKD3*	S	WNL	MD
1061	1q43 dup	Chr1: 239.34–240.99 Mb	1.65	Inh (M)	*RGS7*	C	WNL	WNL
1007^a^	6p21 dup	Chr6: 44.99–46.12 Mb	1.10	Inh (M)	*RUNX2*	M	WNL	WNL
1019^a^	6p21 dup	Chr6: 44.99–46.12 Mb	1.10	Inh (P^b^)	*RUNX2*	M	WNL	WNL
2076	2q14 dup	Chr2: 115.97–116.70 Mb	0.73	Unk	*DPP10*^*+*^	S	A	WNL
1012	17q25 del	Chr17: 78.05–78.65 Mb	0.60	Inh (M)	*WDR45L*, *TBCD*	M	MD	MD
1063	6q26 del	Chr6: 162.84–163.46 Mb	0.62	Unk	*PARK2*, *PACRG*	S	WNL	MD
2003	11q25 dup	Chr11: 130.22–130.67 Mb	0.45	Unk	*SNX19*^*++*^	C	MD	MD
1020	2p21 dup	Chr2: 45.75–46.15 Mb	0.40	Unk	*PRKCE*^*+*^	S	WNL	WNL
2024	7q36 del	Chr7: 158.17–158.33 Mb	0.16	Unk	*ESYT2*^*++*^	M	WNL	WNL
4033	12p12 del	Chr12: 18.12–18.20 Mb	0.08	Unk	*RERGL*^*++*^	S	WNL	WNL
SAG02	9q21 del	Chr9: 74.25–74.60 Mb	0.04	Unk	*TMC1*^*+*^	S	—	—

^+^Gene listed is the only genes with the CNV region and is either partially (^*+*^) or entirely (^*++*^) within CNV; other regions contain genes in addition to those listed.

a Patients 1007 and 1019 are first cousins.

b bInheritance for patient 1019 is inferred to be paternal (Fig. 1).

c Bayley Scales of Infant Development-II index scores: A, accelerated; WNL, within normal limits (BSID-II score 85–114); MD, mildly delayed (BSID-II score 70–84); SD, significantly delayed (BSID-II score 69 or below) [Bayley, 1993].

### Large, Inherited Events in Six Patients

#### 6p21 duplication, 1.1 Mb

We identified a heterozygous inverted duplication of 6p12.3-p21.1 encompassing the *RUNX2* gene in two individuals with metopic synostosis (Fig. 1). Review of the patient database revealed that the two patients were first cousins. Patient 1007 presented with trigonocephaly in infancy; metopic synostosis was confirmed by CT at 1 month of age and repaired at 10 months of age. She was also noted to have a ventricular septal defect. At 36 months, she had normal mental, motor, and language development. At 8-year old, she is known to be missing several permanent teeth. Patient 1019, a maternal first cousin of patient 1007, also had metopic synostosis, noted at 4 months of age. He had a normal cardiac exam; at 7 months he had normal cognitive development and mildly delayed motor development. He is also reported to have hypodontia. We confirmed the presence of the same duplication in the mother of 1007; DNA from the father of 1019 was unavailable for confirmation, though he would be an obligate carrier of the same duplication (we verified that the mother of 1019 does not have the duplication). Both carrier parents have hypodontia by report. The grandfather of 1007 and 1019 is described as having an abnormal head shape with a narrow forehead and several missing teeth; DNA was not available from this individual.

#### 9q22 deletion, 3.92 Mb

**FIG. 2:**
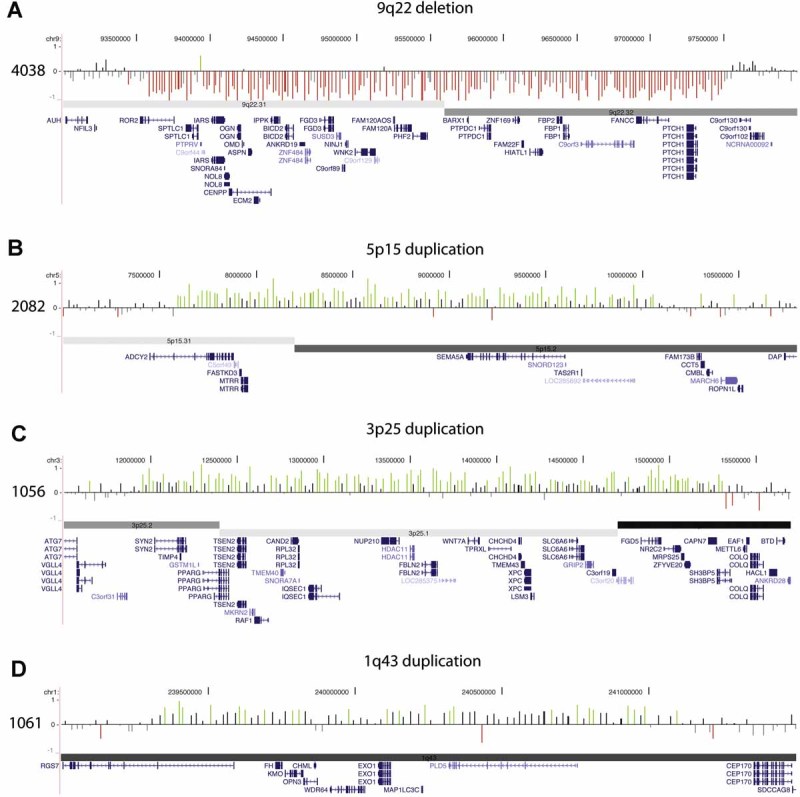
Oligonucleotide array CGH results for (**A**) case 4038 with 3.9 Mb deletion of 9q22; (**B**) case 2082 with 2.5 Mb duplication of 5p15, (**C**) case 1056 with 3.3 Mb duplication of 3p25 and (**D**) case 1061 with a 1.6 Mb duplication of 1q43. Results are presented as in Figure 1.

We identified one individual with metopic synostosis and a large heterozygous deletion of chromosome 9q22 encompassing 33 genes (patient 4038; Fig. 2A). Since entering the study as an infant, this patient has had severe developmental delay. There are several reports in the literature of similar interstitial deletions of 9q [Boonen et al., 2005; Redon et al., 2006; Nowakowska et al., 2007]. Two patients with large deletions of 9q22 were both described with trigonocephaly [Redon et al., 2006]. The deletion in our patient, which is smaller than the previously reported deletions, supports the hypothesis that there is a gene influencing cranial development and narrows the critical region. Although parental DNA was unavailable for analysis, all cases reported to date are de novo.

#### 5p15 duplication, 2.47 Mb

Patient 2082 was born with sagittal synostosis and carries a 2.46 Mb interstitial duplication of chromosome 5p15.2-p15.31 involving seven genes (Fig. 2B). The duplication is also present in the patient's unaffected mother. Cognitive development was normal, but motor development was mildly delayed at 18 months. The duplication has not been reported in 2,493 controls or in the database of genomic variants (DGV). The duplicated region includes *FASTKD3*, a fast kinase domain gene, and semaphorin 5A, which has a possible role in axon guidance.

#### 3p25 duplication, 3.34 Mb

Patient 1056 was born with sagittal synostosis and has a 3.35 Mb duplication of chromosome 3p25 (Fig. 2C), which is also present in the patient's unaffected father. This patient also had delayed motor development, but normal cognitive and language skills at 37 months. There were several entries in the DGVs (http://projects.tcag.ca/variation/) [Iafrate et al., 2004] within this region, but none that encompass the entire region. Similarly, there were three entries in the DECIPHER database, each smaller and contained within the region duplicated in our patient (DECIPHER cases 615, 952, and 1465). Interestingly, DECIPHER case 1465 is a patient with syndromic craniosynostosis [Jehee et al., 2008] for which the duplication was considered non-pathogenic because it was inherited from a normal parent. The other two DECIPHER entries are reported to have MR; in one case the duplication is inherited, and the other is of unknown inheritance. This region contains several interesting candidate genes including *FBLN2* and *WNT7A*.

#### 1q43 duplication, 1.65 Mb

Patient 1061 was born with unilateral coronal synostosis and had normal motor and cognitive development at 37 months of age. She inherited a 1.65 Mb duplication of chromosome 1q43 from her unaffected mother (Fig. 2D). This region contains nine RefSeq genes, one of which is *RGS7*, a regulator of G-protein signaling. There is only one small DGV entry and no similar duplication is reported in DECIPHER.

In addition to the large events described above, eight patients had a smaller deletion or duplication that has not been previously described in control individuals. The events range from 35 to 730 kb in size, and six of the eight events involve a single gene (Table I).

### Sequence Analysis of *RUNX2*

Due to the identification of two patients harboring duplications involving *RUNX2*, and experimental evidence suggesting that increased expression or gain of function is a plausible mechanism in premature suture fusion, we hypothesized that point mutations in *RUNX2* may be detected in a subset of patients. We screened the RUNX2 coding sequence, intron–exon boundaries and 2 kb of sequence upstream of the start codon in all 186 individuals in an attempt to identify point mutations as a cause of single-suture craniosynostosis. No pathogenic sequence changes were identified (data not shown).

### Expression of *RUNX2*

For the two patients with duplications of *RUNX2*, we evaluated expression levels to determine if increased copy number results in increased transcription. We compared expression levels in osteoblasts from these two individuals to expression levels in osteoblasts from unaffected individuals (n = 6) and individuals with synostosis but without duplication of *RUNX2* (n = 22). We found no statistically significant difference in *RUNX2* expression levels (data not shown).

## DISCUSSION

We evaluated 186 individuals with single-suture craniosynostosis for CNVs in order to identify novel candidate genes for craniosynostosis. Within our cohort, 7.5% of individuals carried a deletion or duplication involving one or more genes that has not been previously reported in unaffected individuals. We identified three individuals with CNVs >2 Mb. Although two of these are inherited, when compared to a large control cohort, we found a slight excess of events >2 Mb [3/186 (1.6%) vs. 8/2,493 (0.3%); *P* = 0.036, Fisher's exact test]. We also note that all of the events >1 Mb disrupted at least one gene. By comparison, only 44% of events >1 Mb in our control set of 2,493 individuals disrupted one or more genes. Although it is possible that gene disruption, in addition to or distinct from gene dosage, may influence phenotypic outcome in craniosynostosis our data did not reach statistical significance for this hypothesis (*P* = 0.06). To date, there is only one published study investigating the role of submicroscopic chromosomal rearrangements in a small series of patients with syndromic craniosynostosis using a variety of methods [Jehee et al., 2008]. That study identified chromosome abnormalities in a large fraction of affected individuals (42%), suggesting that gene dosage may be an important mechanism in craniosynostosis. We note that the fraction of individuals with rare CNVs is substantially smaller than that found by Jehee et al. [2008]. However, the individuals in our cohort—with isolated single-suture craniosynostosis—are probably less severely affected than those in the previous study.

The most intriguing CNV is present in two individuals in our cohort who are first cousins: a 1.1 Mb duplication encompassing the entire *RUNX2* gene. Loss-of-function mutations in *RUNX2* cause the autosomal dominant disorder, cleidocranial dysplasia (OMIM 119600), characterized by dysgenesis (or agenesis) of the clavicles and delayed closure of the anterior fontanelle [Mundlos et al., 1997; Cunningham et al., 2006]. In addition, the majority of affected individuals also have dental abnormalities including supernumerary (extra) permanent teeth in ∼70% of patients. There are many lines of evidence that suggest the duplication of *RUNX2* may be causative in these two individuals including the converse dental phenotype of hypodontia to that seen in individuals with heterozygous *RUNX2* inactivation. *RUNX2* is a pro-osteogenic protein and is considered the principal osteogenic master switch [Lian et al., 2004] as its activity is necessary and sufficient for osteoblast differentiation. *RUNX2* is expressed in fusing cranial sutures and is upregulated in mice with heterozygous knock-in mutations of *Fgfr1* and Pfeiffer-type craniosynostosis [Zhou et al., 2000] and in patients with non-syndromic craniosynostosis [Nacamuli et al., 2003]. RUNX2 protein function is repressed by TWIST1, mutations of which cause Saethre–Chotzen syndrome (OMIM 101400), a craniosynostosis syndrome characterized by coronal synostosis, facial asymmetry, ptosis small ears, and occasional syndactyly [el Ghouzzi et al., 1997; Howard et al., 1997; Krebs et al., 1997]. *TWIST1* mutations have been shown to disrupt the association of TWIST1 and RUNX2, preventing RUNX2 repression [Bialek et al., 2004].

Taken together, these data suggest that increased expression of *RUNX2* is associated with craniosynostosis. To our knowledge, there is only one other report of a patient with a duplication encompassing the entire *RUNX2* gene [Wilkie et al., 2006]. The patient is described with unicoronal synostosis and learning difficulties and has duplication that is significantly larger than the one that we describe (3.4 Mb, [Colella et al., 2007]). However, this additional case report further supports the likely pathogenicity of the duplications in our two affected patients. The mother of 1007 was confirmed to be a carrier of the duplication, and the father of 1019 is presumed to carry the same duplication; neither is known to have had synostosis suggesting incomplete penetrance for that phenotype, but both are reported to have hypodontia. We propose that increased dosage of *RUNX2* leads to susceptibility to premature suture fusion and tooth abnormalities, and additional genetic, epigenetic, or environmental factors influence the final phenotypic outcome.

We evaluated expression of *RUNX2* in osteoblast cells from the two patients with a duplication. Although this study was underpowered due to the availability of only two affected cell lines, the average expression of RUNX2 in these samples was higher than all affected and unaffected cell lines. These data should be interpreted with caution but are supportive of a role of *RUNX2* overexpression as a cause of single-suture craniosynostosis.

One child with sagittal synostosis and mild developmental delay has a 3.34 Mb duplication of chromosome 3p25. Although inherited from his unaffected father, similar duplications have not been reported in large control populations. In addition, Jehee et al. [2008] report a smaller, inherited duplication of the same region on 3p25 in 1/45 individuals with synostosis and additional anomalies. Because the duplication was inherited, the authors concluded that it was not likely to be causative. An alternative explanation is that the region contains one or more dosage-sensitive genes important for craniosynostosis with decreased penetrance. Another patient in our series carries a ∼2.5 Mb duplication of 1q43 that is maternally inherited. We are unaware of any similar duplications in published studies of control individuals. Although phenotypes associated with trisomy 1q43 have been described [Morava et al., 2004], the region that is duplicated in those cases is much larger than the duplication in our patient.

We also identified a 4 Mb deletion of 9q22 in a young girl with metopic synostosis and developmental delay. There are several cases previously reported with overlapping deletions of this region [Boonen et al., 2005; Redon et al., 2006; Nowakowska et al., 2007], and at least two of these cases also had metopic synostosis. Our case, one of the smallest of the deletions reported to date, provides additional support for the presence of a craniosynostosis gene in the region and narrows the critical region. The deletion in our case disrupts the *ROR2* gene and deletes 32 additional genes. There are several genes within the deletion region that could contribute to the synostosis phenotype. Mutations in *ROR2* result in autosomal dominant brachydactyly type B and in the autosomal recessive skeletal dysplasia, Robinow syndrome. Two other genes within the region are involved in bone formation: *OMD* (osteomodulin) and *OGN* (osteoglycin). Importantly, not all individuals with deletions encompassing the region deleted in our patient have craniosynostosis, again suggesting decreased penetrance for the phenotype.

Finally, we also detected a deletion or duplication between 35 and 730 kb in eight individuals. Each of these involves one or several genes, each of which is a potential candidate gene for craniosynostosis (Table I). It will be necessary to evaluate additional affected individuals for deletion, duplication, and point mutations of candidate genes to determine whether they truly play a significant role in craniosynostosis. Likewise, each large event (with the exception of the *RUNX2* duplication) was found in a single individual; identification of recurrent or overlapping events in additional patients will help narrow critical regions and candidate genes. We propose that changes in gene dosage of critical genes, including *RUNX2*, increases susceptibility to premature suture fusion.
